# 3,3′-(*p*-Phenyl­enedimethyl­ene)di-1*H*-imidazol-1-ium bis­(4-nitro­benzoate)–4-nitro­benzoic acid (1/2)

**DOI:** 10.1107/S160053681001929X

**Published:** 2010-05-29

**Authors:** Gui-Ying Dong, Xin-Hua Liu, Tong-Fei Liu, Islam Ullah Khan

**Affiliations:** a College of Chemical Engineering and Biotechnology, Hebei Polytechnic University, Tangshan 063009, People’s Republic of China; b College of Light Industry, Hebei Polytechnic University, Tangshan 063009, People’s Republic of China; c Materials Chemistry Laboratory, Department of Chemistry, Government College University, Lahore 54000, Pakistan

## Abstract

The asymmetric unit of the title compound, C_14_H_16_N_4_^2+^·2C_7_H_4_NO_4_^−^·2C_7_H_5_NO_4_, comprises one-half of the 3,3′-(*p*-phenyl­enedimethyl­ene)di-1*H*-imidazol-1-ium dication, which lies on an inversion centre, one 4-nitro­benzoate anion and one 4-nitro­benzoic acid mol­ecule. In the crystal, the components are linked into a two-dimensional network parallel to (110) by O—H⋯O, N—H⋯O and C—H⋯O hydrogen bonds.

## Related literature

For the synthesis of 1,4-bis­(imidazol-1-ylmeth­yl)benzene, see: Hoskins *et al.* (1997 ▶). For a related structure, see: Chen *et al.* (2010 ▶).
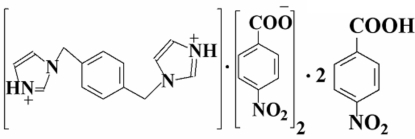


## Experimental

### 

#### Crystal data

C_14_H_16_N_4_^2+^·2C_7_H_4_NO^4−^·2C_7_H_5_NO_4_
*M* *_r_* = 906.77
Triclinic, 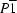

*a* = 7.2659 (15) Å
*b* = 12.689 (3) Å
*c* = 13.028 (3) Å
α = 112.94 (3)°
β = 102.49 (3)°
γ = 101.94 (3)°
*V* = 1021.8 (6) Å^3^
*Z* = 1
Mo *K*α radiation
μ = 0.12 mm^−1^
*T* = 295 K
0.20 × 0.20 × 0.20 mm

#### Data collection

Bruker SMART CCD area-detector diffractometer
Absorption correction: multi-scan (*SADABS*; Sheldrick, 1996 ▶) *T*_min_ = 0.968, *T*_max_ = 0.971
8835 measured reflections
3590 independent reflections
2019 reflections with *I* > 2σ(*I*)
*R*_int_ = 0.066

#### Refinement

*R*[*F* ^2^ > 2σ(*F* ^2^)] = 0.081
*wR*(*F* ^2^) = 0.191
*S* = 1.19
3590 reflections
298 parameters
H-atom parameters constrained
Δρ_max_ = 0.42 e Å^−3^
Δρ_min_ = −0.24 e Å^−3^

### 

Data collection: *SMART* (Bruker, 1998 ▶); cell refinement: *SAINT* (Bruker, 1999 ▶); data reduction: *SAINT*; program(s) used to solve structure: *SHELXS97* (Sheldrick, 2008 ▶); program(s) used to refine structure: *SHELXL97* (Sheldrick, 2008 ▶); molecular graphics: *SHELXTL* (Sheldrick, 2008 ▶); software used to prepare material for publication: *SHELXTL*.

## Supplementary Material

Crystal structure: contains datablocks I, global. DOI: 10.1107/S160053681001929X/ci5093sup1.cif

Structure factors: contains datablocks I. DOI: 10.1107/S160053681001929X/ci5093Isup2.hkl

Additional supplementary materials:  crystallographic information; 3D view; checkCIF report

## Figures and Tables

**Table 1:** Hydrogen-bond geometry (Å, °)

*D*—H⋯*A*	*D*—H	H⋯*A*	*D*⋯*A*	*D*—H⋯*A*
O7—H2*A*⋯O3^i^	0.85	2.57	3.167 (5)	128
O7—H2*A*⋯O4^i^	0.85	1.65	2.494 (5)	173
N4—H4*A*⋯O8	0.86	2.03	2.690 (5)	133
C15—H15⋯O3	0.93	2.23	3.073 (7)	150
C17—H17⋯O5^ii^	0.93	2.46	3.228 (7)	140
C21—H21⋯O3^iii^	0.93	2.46	3.321 (6)	154

Symmetry codes: (i) 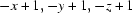; (ii) 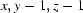; (iii) 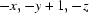.

**Fig. 1.**
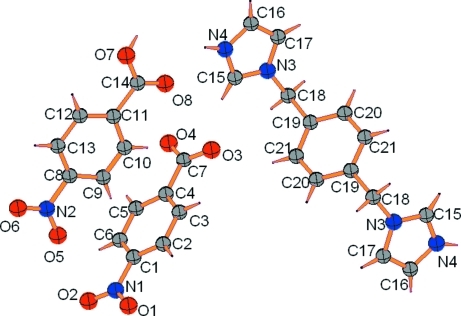
The asymmetric unit of the title compound, showing the atomic numbering and 30% probability displacement ellipsoids. Atoms labelled with the suffix A are generated by the symmetry operation (-x, 1-y, -z).

## supplementary crystallographic information

### Comment

Over the past few years, efforts have been focused on the investigation of coordination polymers with flexible ligands. Di-imidazole flexbile ligands such as 1,4-bis(1H-imidazol-1-yl)methylbenzene (bix) find numerous applications in constructing metal-organic coordination polymers as they can rotate freely about methylene carbon atoms to adjust to the coordination environment. We report here the crystal structure of the title compound.

The asymmetric unit comprises one-half of a bix^2+^ dication lying on an inversion centre, one 4-nitrobenzoate anion and one neutral 4-nitrobenzoic acid molecule (Fig. 1). Bond distances and angles are normal (Chen *et al.*, 2010).

In the crystal structure, the dications, anions and neutral 4-nitrobenzoic acid molecule are interlinked by O—H···O, N—H···O and C—H···O hydrogen bonds (Table 1), forming a two-dimensional hydrogen-bonded network parallel to the (110).

### Experimental

1,4-Bis(imidazol-1-ylmethyl)benzene (bix) was prepared according to a literature method (Hoskins *et al.*, 1997). 1:4 molar amounts of bix (23.8 mg, 0.1 mmol) and 4-nitrobenzoic acid (66.9 mg , 0.4 mmol) were dissolved in 95% ethanol (30 ml). The mixture was stirred and refluxed for 1 h and then filtered. The resulting colourless solution was allowed to stand in air for two weeks. Colourless crystals of the title compound suitable for X-ray diffraction analysis were obtained by slow evaporation of the solution.

### Refinement

H atoms were positioned geometrically [O–H = 0.85 Å, N–H = 0.86 Å and C–H = 0.93 or 0.97 Å] and refined using a riding model, with U_iso_(H) = 1.5U_eq_(O) and 1.2U_eq_(C,N).

### Crystal data

**Table:**

C_14_H_16_N_4_^2+^·2C_7_H_4_NO^4^^−^·2C_7_H_5_NO_4_	*Z* = 1
*M**_r_* = 906.77	*F*(000) = 470
Triclinic, *P*1	*D*_x_ = 1.474 Mg m^−^^3^
Hall symbol: -P 1	Mo *K*α radiation, λ = 0.71073 Å
*a* = 7.2659 (15) Å	Cell parameters from 3889 reflections
*b* = 12.689 (3) Å	θ = 4.6–22.7°
*c* = 13.028 (3) Å	µ = 0.12 mm^−^^1^
α = 112.94 (3)°	*T* = 295 K
β = 102.49 (3)°	Prism, colourless
γ = 101.94 (3)°	0.20 × 0.20 × 0.20 mm
*V* = 1021.8 (6) Å^3^	

### Data collection

**Table:**

Bruker SMART CCD area-detector diffractometer	3590 independent reflections
Radiation source: fine–focus sealed tube	2019 reflections with *I* > 2σ(*I*)
graphite	*R*_int_ = 0.066
φ and ω scans	θ_max_ = 25.0°, θ_min_ = 3.0°
Absorption correction: multi-scan (*SADABS*; Sheldrick, 1996)	*h* = −8→8
*T*_min_ = 0.968, *T*_max_ = 0.971	*k* = −15→15
8835 measured reflections	*l* = −15→15

### Refinement

**Table:**

Refinement on *F*^2^	Primary atom site location: structure-invariant direct methods
Least-squares matrix: full	Secondary atom site location: difference Fourier map
*R*[*F*^2^ > 2σ(*F*^2^)] = 0.081	Hydrogen site location: inferred from neighbouring sites
*wR*(*F*^2^) = 0.191	H-atom parameters constrained
*S* = 1.19	*w* = 1/[σ^2^(*F*_o_^2^) + (0.0648*P*)^2^ + 0.2399*P*] where *P* = (*F*_o_^2^ + 2*F*_c_^2^)/3
3590 reflections	(Δ/σ)_max_ = 0.001
298 parameters	Δρ_max_ = 0.42 e Å^−^^3^
0 restraints	Δρ_min_ = −0.24 e Å^−^^3^

### Special details

**Table:**

Geometry. All esds (except the esd in the dihedral angle between two l.s. planes) are estimated using the full covariance matrix. The cell esds are taken into account individually in the estimation of esds in distances, angles and torsion angles; correlations between esds in cell parameters are only used when they are defined by crystal symmetry. An approximate (isotropic) treatment of cell esds is used for estimating esds involving l.s. planes.
Refinement. Refinement of F^2^ against ALL reflections. The weighted R-factor wR and goodness of fit S are based on F^2^, conventional R-factors R are based on F, with F set to zero for negative F^2^. The threshold expression of F^2^ > 2sigma(F^2^) is used only for calculating R-factors(gt) etc. and is not relevant to the choice of reflections for refinement. R-factors based on F^2^ are statistically about twice as large as those based on F, and R- factors based on ALL data will be even larger.

### Fractional atomic coordinates and isotropic or equivalent isotropic displacement parameters (Å^2^)

**Table:**

	*x*	*y*	*z*	*U*_iso_*/*U*_eq_	
O7	0.1748 (5)	0.3773 (3)	0.6201 (3)	0.0628 (9)	
O8	0.2413 (5)	0.4626 (3)	0.5069 (3)	0.0636 (9)	
N4	0.2722 (5)	0.3310 (3)	0.2960 (3)	0.0468 (9)	
H4A	0.2596	0.3328	0.3607	0.056*	
N3	0.3203 (4)	0.3884 (3)	0.1672 (3)	0.0341 (8)	
C11	0.2099 (5)	0.5846 (3)	0.6892 (3)	0.0346 (10)	
C12	0.1960 (6)	0.5971 (4)	0.7973 (3)	0.0378 (10)	
H12	0.1787	0.5305	0.8124	0.045*	
C20	0.0546 (6)	0.4031 (4)	−0.0614 (3)	0.0417 (11)	
H20	0.0910	0.3371	−0.1031	0.050*	
C10	0.2349 (6)	0.6835 (4)	0.6673 (4)	0.0454 (11)	
H10	0.2447	0.6753	0.5947	0.054*	
C21	−0.1173 (6)	0.4176 (4)	−0.1146 (4)	0.0417 (11)	
H21	−0.1952	0.3621	−0.1918	0.050*	
C13	0.2074 (6)	0.7074 (4)	0.8830 (4)	0.0426 (11)	
H13	0.1981	0.7160	0.9558	0.051*	
C17	0.2842 (6)	0.2657 (4)	0.1182 (4)	0.0397 (10)	
H17	0.2808	0.2161	0.0427	0.048*	
C8	0.2328 (6)	0.8040 (4)	0.8581 (4)	0.0423 (11)	
C14	0.2070 (6)	0.4663 (4)	0.5962 (4)	0.0417 (11)	
C19	0.1743 (6)	0.4850 (4)	0.0531 (3)	0.0359 (10)	
C9	0.2457 (6)	0.7939 (4)	0.7505 (4)	0.0510 (12)	
H9	0.2612	0.8603	0.7351	0.061*	
N2	0.2409 (6)	0.9230 (4)	0.9474 (4)	0.0642 (12)	
C15	0.3111 (6)	0.4252 (4)	0.2754 (4)	0.0405 (10)	
H15	0.3291	0.5044	0.3279	0.049*	
O6	0.2194 (6)	0.9302 (3)	1.0400 (3)	0.0946 (14)	
C16	0.2548 (6)	0.2308 (4)	0.1995 (4)	0.0445 (11)	
H16	0.2277	0.1525	0.1913	0.053*	
O5	0.2639 (7)	1.0065 (3)	0.9231 (4)	0.1088 (16)	
C18	0.3622 (6)	0.4672 (4)	0.1113 (4)	0.0472 (11)	
H18A	0.4226	0.4318	0.0525	0.057*	
H18B	0.4567	0.5452	0.1706	0.057*	
C4	0.4933 (6)	0.8837 (3)	0.5529 (3)	0.0360 (10)	
O3	0.4409 (5)	0.7026 (3)	0.3853 (3)	0.0731 (11)	
O2	0.4178 (6)	1.2399 (3)	0.8522 (3)	0.0791 (11)	
O4	0.7429 (5)	0.7925 (3)	0.5183 (3)	0.0700 (10)	
C1	0.3648 (7)	1.0618 (4)	0.6869 (4)	0.0423 (11)	
N1	0.2961 (7)	1.1562 (4)	0.7606 (4)	0.0616 (11)	
C5	0.6275 (6)	0.9830 (3)	0.6549 (3)	0.0417 (11)	
H5	0.7610	0.9887	0.6779	0.050*	
C3	0.2940 (6)	0.8758 (4)	0.5190 (3)	0.0432 (11)	
H3	0.2041	0.8099	0.4503	0.052*	
C2	0.2281 (7)	0.9650 (4)	0.5863 (4)	0.0473 (11)	
H2	0.0947	0.9596	0.5642	0.057*	
C6	0.5638 (7)	1.0733 (4)	0.7224 (4)	0.0451 (11)	
H6	0.6533	1.1403	0.7903	0.054*	
C7	0.5608 (8)	0.7845 (4)	0.4785 (4)	0.0470 (11)	
O1	0.1202 (7)	1.1467 (4)	0.7276 (4)	0.1083 (15)	
H2A	0.2107	0.3206	0.5773	0.162*	

### Atomic displacement parameters (Å^2^)

**Table:**

	*U*^11^	*U*^22^	*U*^33^	*U*^12^	*U*^13^	*U*^23^
O7	0.086 (3)	0.0432 (19)	0.072 (2)	0.0297 (18)	0.0469 (19)	0.0238 (18)
O8	0.086 (3)	0.065 (2)	0.0381 (19)	0.0274 (19)	0.0290 (18)	0.0162 (17)
N4	0.048 (2)	0.062 (3)	0.035 (2)	0.0202 (19)	0.0143 (17)	0.025 (2)
N3	0.035 (2)	0.038 (2)	0.032 (2)	0.0177 (16)	0.0105 (15)	0.0157 (17)
C11	0.028 (2)	0.039 (2)	0.032 (2)	0.0081 (18)	0.0080 (18)	0.013 (2)
C12	0.039 (3)	0.042 (3)	0.041 (3)	0.016 (2)	0.019 (2)	0.023 (2)
C20	0.049 (3)	0.041 (3)	0.043 (3)	0.023 (2)	0.018 (2)	0.021 (2)
C10	0.058 (3)	0.047 (3)	0.034 (3)	0.017 (2)	0.017 (2)	0.019 (2)
C21	0.049 (3)	0.038 (3)	0.032 (2)	0.013 (2)	0.008 (2)	0.013 (2)
C13	0.041 (3)	0.053 (3)	0.034 (2)	0.017 (2)	0.016 (2)	0.016 (2)
C17	0.045 (3)	0.034 (2)	0.037 (3)	0.020 (2)	0.012 (2)	0.011 (2)
C8	0.038 (3)	0.036 (2)	0.041 (3)	0.013 (2)	0.009 (2)	0.007 (2)
C14	0.029 (2)	0.045 (3)	0.042 (3)	0.009 (2)	0.008 (2)	0.013 (2)
C19	0.036 (3)	0.040 (2)	0.037 (3)	0.008 (2)	0.011 (2)	0.025 (2)
C9	0.064 (3)	0.043 (3)	0.053 (3)	0.019 (2)	0.020 (2)	0.026 (3)
N2	0.065 (3)	0.050 (3)	0.059 (3)	0.022 (2)	0.013 (2)	0.008 (3)
C15	0.033 (3)	0.040 (3)	0.035 (3)	0.015 (2)	0.0044 (19)	0.006 (2)
O6	0.138 (4)	0.083 (3)	0.046 (2)	0.055 (3)	0.026 (2)	0.006 (2)
C16	0.046 (3)	0.034 (3)	0.050 (3)	0.014 (2)	0.010 (2)	0.018 (2)
O5	0.177 (5)	0.046 (2)	0.115 (4)	0.046 (3)	0.072 (3)	0.029 (2)
C18	0.041 (3)	0.054 (3)	0.053 (3)	0.015 (2)	0.014 (2)	0.032 (2)
C4	0.053 (3)	0.025 (2)	0.032 (2)	0.012 (2)	0.015 (2)	0.0134 (19)
O3	0.082 (3)	0.047 (2)	0.051 (2)	0.0251 (18)	−0.0019 (19)	−0.0063 (17)
O2	0.091 (3)	0.054 (2)	0.066 (2)	0.023 (2)	0.032 (2)	−0.001 (2)
O4	0.057 (2)	0.047 (2)	0.076 (2)	0.0193 (17)	0.0156 (19)	0.0005 (18)
C1	0.056 (3)	0.036 (2)	0.046 (3)	0.021 (2)	0.025 (2)	0.021 (2)
N1	0.067 (3)	0.049 (3)	0.065 (3)	0.023 (2)	0.035 (3)	0.015 (2)
C5	0.044 (3)	0.033 (2)	0.042 (3)	0.010 (2)	0.010 (2)	0.014 (2)
C3	0.050 (3)	0.036 (3)	0.033 (2)	0.009 (2)	0.004 (2)	0.013 (2)
C2	0.047 (3)	0.044 (3)	0.043 (3)	0.013 (2)	0.011 (2)	0.016 (2)
C6	0.059 (3)	0.033 (2)	0.034 (2)	0.012 (2)	0.014 (2)	0.009 (2)
C7	0.066 (4)	0.028 (2)	0.042 (3)	0.012 (2)	0.015 (3)	0.014 (2)
O1	0.072 (3)	0.086 (3)	0.129 (4)	0.040 (2)	0.037 (3)	0.003 (3)

### Geometric parameters (Å, °)

**Table:**

O7—C14	1.274 (5)	C8—N2	1.484 (5)
O7—H2A	0.85	C19—C21^i^	1.387 (5)
O8—C14	1.227 (5)	C19—C18	1.519 (5)
N4—C15	1.313 (5)	C9—H9	0.93
N4—C16	1.354 (5)	N2—O5	1.210 (5)
N4—H4A	0.86	N2—O6	1.220 (5)
N3—C15	1.324 (5)	C15—H15	0.93
N3—C17	1.373 (5)	C16—H16	0.93
N3—C18	1.469 (5)	C18—H18A	0.97
C11—C10	1.378 (5)	C18—H18B	0.97
C11—C12	1.383 (5)	C4—C5	1.389 (5)
C11—C14	1.510 (5)	C4—C3	1.389 (5)
C12—C13	1.380 (5)	C4—C7	1.509 (6)
C12—H12	0.93	O3—C7	1.224 (5)
C20—C21	1.376 (5)	O2—N1	1.225 (5)
C20—C19	1.385 (5)	O4—C7	1.279 (5)
C20—H20	0.93	C1—C2	1.374 (6)
C10—C9	1.370 (6)	C1—C6	1.377 (6)
C10—H10	0.93	C1—N1	1.472 (5)
C21—C19^i^	1.387 (5)	N1—O1	1.221 (5)
C21—H21	0.93	C5—C6	1.382 (5)
C13—C8	1.373 (5)	C5—H5	0.93
C13—H13	0.93	C3—C2	1.382 (5)
C17—C16	1.336 (5)	C3—H3	0.93
C17—H17	0.93	C2—H2	0.93
C8—C9	1.382 (6)	C6—H6	0.93
			
C14—O7—H2A	111.6	O5—N2—O6	123.9 (4)
C15—N4—C16	109.4 (3)	O5—N2—C8	118.0 (5)
C15—N4—H4A	125.3	O6—N2—C8	118.1 (5)
C16—N4—H4A	125.3	N4—C15—N3	108.4 (4)
C15—N3—C17	108.0 (3)	N4—C15—H15	125.8
C15—N3—C18	125.0 (3)	N3—C15—H15	125.8
C17—N3—C18	127.0 (3)	C17—C16—N4	107.0 (4)
C10—C11—C12	119.4 (4)	C17—C16—H16	126.5
C10—C11—C14	119.0 (4)	N4—C16—H16	126.5
C12—C11—C14	121.6 (4)	N3—C18—C19	111.7 (3)
C13—C12—C11	120.7 (4)	N3—C18—H18A	109.3
C13—C12—H12	119.7	C19—C18—H18A	109.3
C11—C12—H12	119.7	N3—C18—H18B	109.3
C21—C20—C19	121.1 (4)	C19—C18—H18B	109.3
C21—C20—H20	119.4	H18A—C18—H18B	107.9
C19—C20—H20	119.4	C5—C4—C3	119.5 (4)
C9—C10—C11	121.2 (4)	C5—C4—C7	121.0 (4)
C9—C10—H10	119.4	C3—C4—C7	119.6 (4)
C11—C10—H10	119.4	C2—C1—C6	122.6 (4)
C20—C21—C19^i^	120.0 (4)	C2—C1—N1	118.9 (4)
C20—C21—H21	120.0	C6—C1—N1	118.5 (4)
C19^i^—C21—H21	120.0	O1—N1—O2	123.3 (4)
C8—C13—C12	118.3 (4)	O1—N1—C1	118.3 (4)
C8—C13—H13	120.8	O2—N1—C1	118.4 (5)
C12—C13—H13	120.8	C6—C5—C4	120.4 (4)
C16—C17—N3	107.1 (4)	C6—C5—H5	119.8
C16—C17—H17	126.4	C4—C5—H5	119.8
N3—C17—H17	126.4	C2—C3—C4	120.7 (4)
C13—C8—C9	122.2 (4)	C2—C3—H3	119.7
C13—C8—N2	119.5 (4)	C4—C3—H3	119.7
C9—C8—N2	118.2 (4)	C1—C2—C3	118.4 (4)
O8—C14—O7	124.9 (4)	C1—C2—H2	120.8
O8—C14—C11	119.4 (4)	C3—C2—H2	120.8
O7—C14—C11	115.7 (4)	C1—C6—C5	118.5 (4)
C20—C19—C21^i^	118.8 (4)	C1—C6—H6	120.7
C20—C19—C18	120.6 (4)	C5—C6—H6	120.7
C21^i^—C19—C18	120.5 (4)	O3—C7—O4	124.3 (4)
C10—C9—C8	118.2 (4)	O3—C7—C4	119.0 (5)
C10—C9—H9	120.9	O4—C7—C4	116.7 (4)
C8—C9—H9	120.9		

Symmetry codes: (i) −*x*, −*y*+1, −*z*.

### Hydrogen-bond geometry (Å, °)

**Table:**

*D*—H···*A*	*D*—H	H···*A*	*D*···*A*	*D*—H···*A*
O7—H2A···O3^ii^	0.85	2.57	3.167 (5)	128
O7—H2A···O4^ii^	0.85	1.65	2.494 (5)	173
N4—H4A···O8	0.86	2.03	2.690 (5)	133
C15—H15···O3	0.93	2.23	3.073 (7)	150
C17—H17···O5^iii^	0.93	2.46	3.228 (7)	140
C21—H21···O3^i^	0.93	2.46	3.321 (6)	154

Symmetry codes: (ii) −*x*+1, −*y*+1, −*z*+1;  (iii) *x*, *y*−1, *z*−1; (i) −*x*, −*y*+1, −*z*.
